# Quantum computation based on photonic systems with two degrees of freedom assisted by the weak cross-Kerr nonlinearity

**DOI:** 10.1038/srep29939

**Published:** 2016-07-18

**Authors:** Ming-Xing Luo, Hui-Ran Li, Hong Lai

**Affiliations:** 1 Information Security and National Computing Grid Laboratory, School of Information Science and Technology, Southwest Jiaotong University, Chengdu 610031, China; 2 Department of Physics, University of Michigan, Ann Arbor, MI 48109, USA; 3 School of Computer and Information Science, Southwest University, Chongqing 400715, China

## Abstract

Most of previous quantum computations only take use of one degree of freedom (DoF) of photons. An experimental system may possess various DoFs simultaneously. In this paper, with the weak cross-Kerr nonlinearity, we investigate the parallel quantum computation dependent on photonic systems with two DoFs. We construct nearly deterministic controlled-not (CNOT) gates operating on the polarization spatial DoFs of the two-photon or one-photon system. These CNOT gates show that two photonic DoFs can be encoded as independent qubits without auxiliary DoF in theory. Only the coherent states are required. Thus one half of quantum simulation resources may be saved in quantum applications if more complicated circuits are involved. Hence, one may trade off the implementation complexity and simulation resources by using different photonic systems. These CNOT gates are also used to complete various applications including the quantum teleportation and quantum superdense coding.

From the quantum circuit model^1^, quantum controlled gates^2^,^3^ play key roles for various quantum applications^4^,^5^,^6^,^7^. It has shown that two-qubit gates, especially the CNOT gate and single-qubit gates are universal for synthesizing quantum tasks based on multiple qubits^2^,^3^,^8^. The pioneer model^9^ takes use of single photon sources, linear optical elements including feed forward, and single photon detectors to realize the CNOT gate with the maximum probability of 3/4^10^. With this standard model, various schemes are proposed to implement the CNOT gate^11^,^12^,^13^,^14^,^15^ and controlled-phase gate^16^,^17^. Although their upper bounds of the success probability are not thought to be tight^18^, however, it has shown that near deterministic gates are impossible using only linear optical elements. Moreover, the multiple-qubit based quantum tasks may be inefficient when lots of probabilistic gates are involved. For an example, the qubit flip coding with three qubits may be constructed using ten CNOT gates (four CNOT gates and one Toffoli gate^19^) and some single-qubit gates^20^, its success probability is only (3/4)^10^ = 5.6 × 10^−2^ with the maximum probability of a CNOT^10^. Hence, more efficient or deterministic gates should be proposed by relaxing constraints in the standard model^9^. Fortunately, with the weak cross-Kerr nonlinearity, a nearly deterministic CNOT gate^21^,^22^ and multiple-qubit logic gates such as Fredkin gate, Toffoli gate, arbitrary controlled-*U* gate^23^ have been proposed on the polarization DoF. These controlled gates are also implemented using different physical systems such as the ion trap^24^,^25^, atom^26^,^27^, and nuclear magnetic resonance^28^,^29^.

Previous implementations of controlled gates have focused on the systems with only one DoF^9^,^11^,^12^,^13^,^14^,^15^,^22^,^23^. Controlled logic gates are always realized on the polarization DoF using auxiliary spatial DoFs^12^,^13^,^14^,^22^,^23^ or auxiliary polarized photons^11^,^15^. If two DoFs are independently used for encoding different information, their conversions may cause confusions in large-scale quantum applications such as the Shor’s algorithm. Moreover, an experimental system may possess various independent DoFs simultaneously. Different DoFs of physical system may be useful in various quantum applications^30^. Recent experiment shows that quantum information may be transferred from the polarization DoF of one photon to the orbital angular momentum of the other photon^31^. By using a hyper-entangled photon pair (the simultaneous entanglement in more than one DoF), Wang *et al*.^32^ have experimentally teleported a photon with the spin angular momentum and orbital angular momentum DoFs while Graham *et al*.^33^ teleported a specific photon of two DoFs with only phase information. Here, the hyperentanglement^34^,^35^,^36^ such as polarization momentum, polarization-time-bin, and polarization- and spatial modes-energy-time can be used to assist the Bell-state discrimination^37^,^38^,^39^,^40^,^41^,^42^,^43^,^44^,^45^,^46^,^47^.

Motivated by the recent experiments^30^,^31^,^32^,^33^ and usefulness of different photonic DoFs^34^,^35^,^36^,^37^,^38^,^39^,^40^,^41^,^42^,^43^,^44^,^45^,^46^, in this paper, we consider the controlled gates on photonic system with two DoFs assisted by the weak cross-Kerr nonlinearity^22^,^23^,^46^,^47^,^48^,^49^,^50^,^51^,^52^,^53^. Different from previous schemes on photonic systems with the polarization DoF^11^,^12^,^13^,^14^,^15^,^22^,^23^, where another DoF is used to assist quantum logic gates, we investigate the photonic quantum computation using two DoFs as simultaneous encoding qubits. To show the independence of two photonic DoFs in each quantum task, from the quantum circuit model the CNOT gate will be implemented on all the combinations of the polarization and spatial DoFs of the two-photon or one-photon system. This is beyond previous CNOT gates on the two-photon system with one DoF^11^,^12^,^13^,^14^,^15^,^22^,^23^. By exploiting the weak cross-Kerr nonlinearity^49^,^50^,^51^,^52^,^53^, all of controlled gates are nearly deterministic without auxiliary DoFs^11^,^15^. In contrast to the hybrid CNOT gates on the photon and stationary electron spins in quantum dots^54^,^55^, our CNOT gates are realized on photonic systems. Our results are also different from previous controlled gates^54^,^55^,^56^,^57^,^58^,^59^, where a CNOT gate is only considered in the same DoF of two photons assisted by a double-sided quantum dot-cavity system^54^,^55^ or one-sided quantum dot-cavity system^56^,^57^,^58^,^59^. Our theoretical results show that two DoFs of a photon system can be independently and simultaneously encoded in each quantum task. With these constructions, one half of quantum resources may be saved for quantum simulations, which are very important in large-scale quantum applications such as the quantum Shor algorithm and network-based quantum communications. To show its applications, we also present faithful teleportation of arbitrary *n*-photon and quantum superdense coding.

## Results

To show the encoding independence of the polarization and spatial DoFs of a photon for any quantum tasks, it is necessary to show that all *n*-qubit quantum operations may be realized on these DoFs. From the universality of the CNOT gate and single-qubit operations in the quantum logic^2^,^3^,^8^, it only needs to consider the CNOT gate on all the combinations of two DoFs of photonic systems. From different roles of two DoFs, six CNOT gates should be implemented, i.e., four CNOT gates on the two-photon system (each DoF of one photon is used) and two CNOT gates on the one-photon system. None of these gates require switching these DoFs during the simulations.

Before expounding our schemes of the CNOT gate, we first introduce the weak cross-Kerr nonlinearity^21^,^22^,^23^,^49^,^50^,^51^,^52^. Given a signal field |*n*_*a*_〉 and a probe beam |*α*〉, after photons passing through the cross-Kerr medium, the joint state of the combined system will be


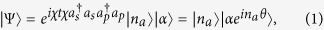


where *θ* = *χt* and *t* is the interaction time. Previous works indicated that a cross-Kerr medium and a coherent state can be used to implement the CNOT gate^22^,^23^ and single-photon logic gates with minimal sources^51^ and Toffoli gate^52^, and complete entanglement purification and concentration^45^,^46^,^47^,^48^; generating high-quality entanglement^49^,^50^ and qubits^60^,^61^,^62^.

### CNOT gate on the polarization DoFs of two photons

Suppose that two photons are initially prepared in the state


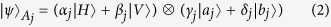


for the simplicity of schematic representation, where {*a*_*j*_, *b*_*j*_} is the basis of the spatial DoF (the paraxial spatial modes (Laguerre-Gauss) carrying −*ħ* and *ħ* orbital angular momentum) of photon *A*_*j*_. The same results can be followed for general forms of a two-photon system. Our consideration in this subsection is to realize the CNOT gate on the polarization DoFs of two photons.

**Figure 1:**
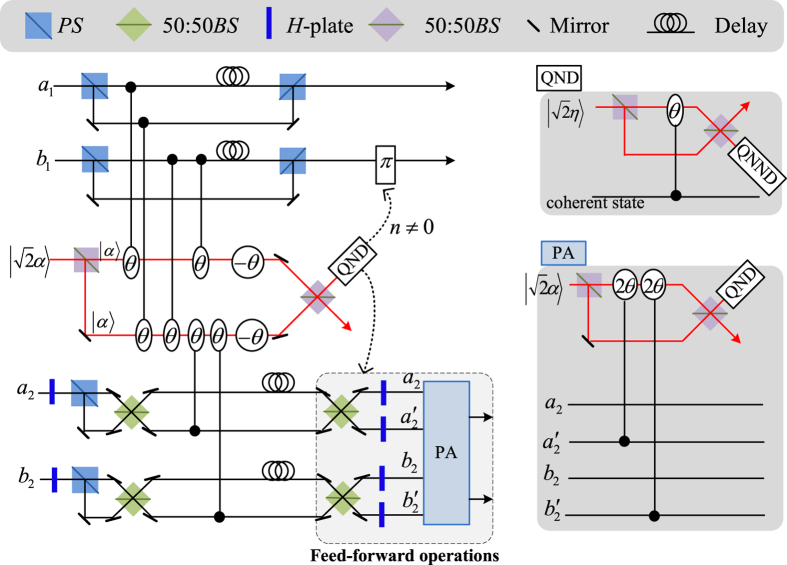
Schematic CNOT gate on the polarization DoFs of two photons. PS denotes a beam splitter to transmit |*H*〉 and reflect |*V*〉 of an input photon. BS denotes a 50:50 polarizing beam splitter to realize 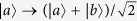 and 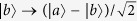 on two spatial modes *a* and *b* of an input photon. An auxiliary probe beam is in the coherent state 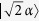
49,51. Another BS denotes a 50:50 polarizing beam splitter to implement the transformation 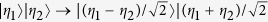 for the auxiliary coherent photons. *H*-plate denotes a half-wave plate to perform the Hadamard operation 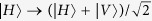 and 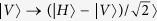. QND denotes quantum nondemolition module^53^ of the coherent photons. PNND denotes the photon number non-resolving detector. PA denotes a quantum parity gate for one photon with four spatial modes.

Schematic circuit is shown in Fig. 1 using the double cross-phase modulation technique^49^,^51^,^52^,^53^ to avoid an impractical interacted-phase shift −*θ*^21^,^22^. The controlling photon *A*_1_ from each mode passes through a *PS*, interacts with the coherent photons with a phase *θ*, and another *PS*. And then, the photon *A*_2_ passes through a *H* and a *BS*, and interacts with the second coherent pulse with a phase *θ*. Now, two coherent pulses pass through a phase shifter −*θ, BS* and QND (a quantum nondemolition module)^53^. In detail, the joint system of two input photons (*A*_1_ and *A*_2_) and the auxiliary coherent pulse evolves from the initial state 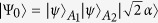 as follows:


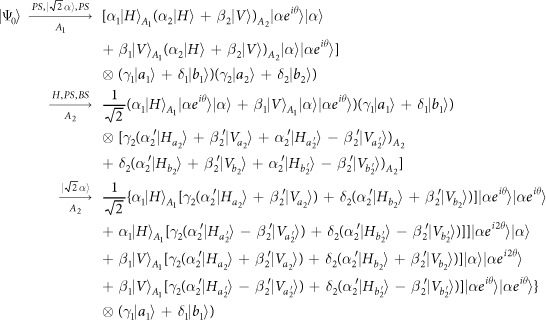



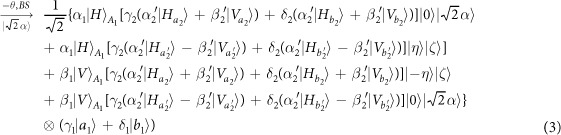


where 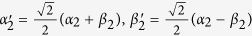, 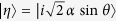 and 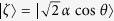.

Due to the quantum noise effect, the Homodyne detection cannot work well as its expected^63^. Afterwards, the quantum nondemolition module is used to discriminate two coherent states. In detail, the projection |*n*〉 〈*n*| is performed on the first qubus beam to get the proper output^53^. If the measurement outcome is *n* = 0, the photonic state in the Eq. (3) collapses into


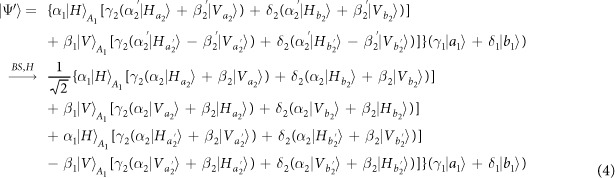


Now, by using a parity analyzer (PA) for the photon *A*_2_, if *n* = 0 for the new Homodyne detection (the photon *A*_2_ passes through the modes *a*_2_ and *b*_2_, see the Method), the state in the Eq. (4) will be


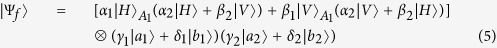


Otherwise, *n* ≠ 0 for the new Homodyne detection (the photon *A*_2_ passes through the modes 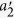 and 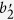, see Method), the state in the Eq. (4) will be |Ψ_*f*_〉 in the Eq. (5) after a Pauli flip *Z* = |*H*〉 〈*H*| − |*V*〉 〈*V*| on the photon *A*_1_. Here, the unmeasured beams in the state 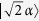 may be reused.

If the measurement outcome satisfies *n* ≠ 0, the photonic state in the Eq. (3) collapses into


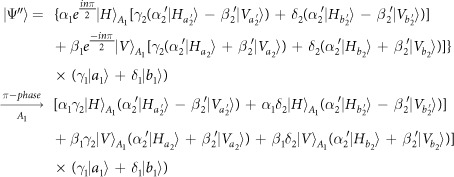



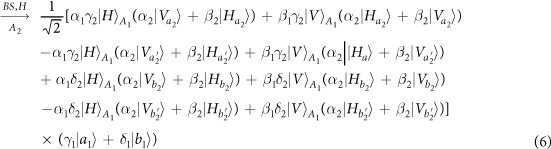


In the follow, using a PA for the photon *A*_2_ (similar projection has been performed for two modes^53^), if the photon *A*_2_ passes through the modes *a*_2_ and *b*_2_ (*n* ≠ 0 for the new Homodyne detection), the state in the Eq. (6) will be |Ψ_*f*_〉 in the Eq. (5). If the photon *A*_2_ passes through the modes 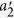 and 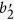 (*n* ≠ 0 for the new Homodyne detection), the state in the Eq. (6) will be |Ψ_*f*_〉 in the Eq. (5) after a Pauli flip *σ*_*z*_ on the photon *A*_1_. The projection |*n*〉 〈*n*| may be approximated by a transition edge sensor-a superconducting microbolometer^49^,^51^. Thus a CNOT gate has been nearly deterministically implemented on the polarization DoFs of two photons. Here, the unmeasured beams in the state 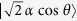 may be reused.

### CNOT gate on the spatial DoFs of two photons

**Figure 2:**
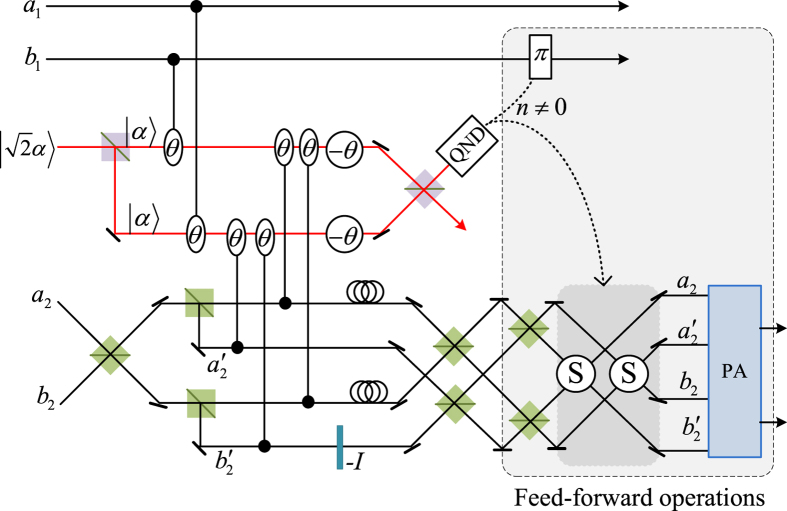
Schematic CNOT gate on the spatial DoFs of two photons. PS, BS, QND and PA are defined in the Fig. 1. −*I* denotes a wave plate to perform the phase operation −|*H*〉 〈*H*| − |*V*〉 〈*V*|. *S* denotes the switching operation (NOT gate) of two spatial modes, which may be realized with two BSs and a waveplate −*I* on the second mode. An auxiliary probe beam is in the coherent state 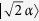.

Our consideration in this subsection is to realize a CNOT gate on the spatial DoFs of two photons. The schematic circuit is shown in Fig. 2. The joint system of two photons *A*_1_ and *A*_2_ and the coherent photon evolve from the initial state |Ψ_0_〉 as follows:


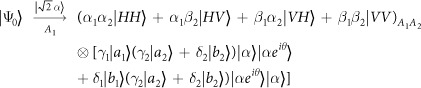



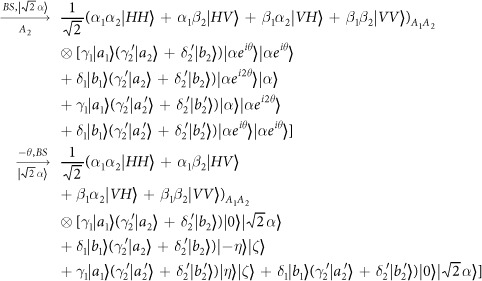



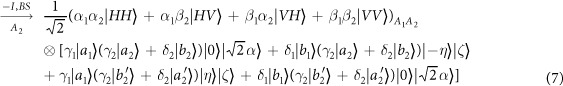


where 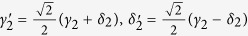, |*η*〉 and |*ζ*〉 are defined in the Eq. (3).

Afterwards, the projection |*n*〉 〈*n*| is performed on the first qubus beam to get the proper output^49^,^51^. If the measurement outcome is *n* = 0, the photonic state in the Eq. (7) collapses into


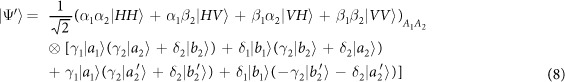


after the photon *A*_2_ passing through a BS. Now, using a PA for the photon *A*_2_, the state in the Eq. (8) will be


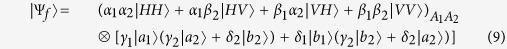


if *n* = 0 for the new Homodyne detection (the photon *A*_2_ passes through the modes *a*_2_ and *b*_2_). When *n* ≠ 0 for the new Homodyne detection (the photon *A*_2_ passes through the modes *a*′_2_ and *b*′_2_), the state in the Eq. (8) may be changed into |Ψ_*f*_〉 in the Eq. (9) using a phase gate −*I* on the photon *A*_1_ from the spatial mode *b*_1_.

If the measurement outcome satisfies *n* ≠ 0, the photonic state in the Eq. (7) collapses into


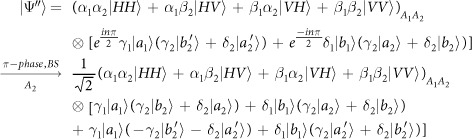



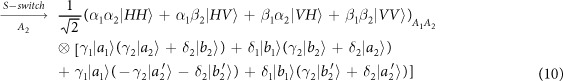


here, each switch operation *S* is a *NOT* = *H* · *Z* · *H* gate of two spatial modes, which may be realized with two BSs and a waveplate −*I* on the second mode. Now, by using PA for the photon *A*_2_, if *n* = 0 for the new Homodyne detection (the photon *A*_2_ passes through the modes *a*_2_ and *b*_2_), the state in the Eq. (10) will be |Ψ_*f*_〉 in the Eq. (9). If *n* ≠ 0 for the new Homodyne detection (the photon *A*_2_ passes through the modes 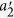 and 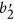), the state in the Eq. (10) will be |Ψ_*f*_〉 in the Eq. (9) using a phase gate −*I* on the photon *A*_1_ from the spatial mode *a*_1_. Thus a CNOT gate has been nearly deterministically implemented on the spatial DoFs of two photons.

### CNOT gate on the polarization-spatial DoFs of a two-photon system

**Figure 3:**
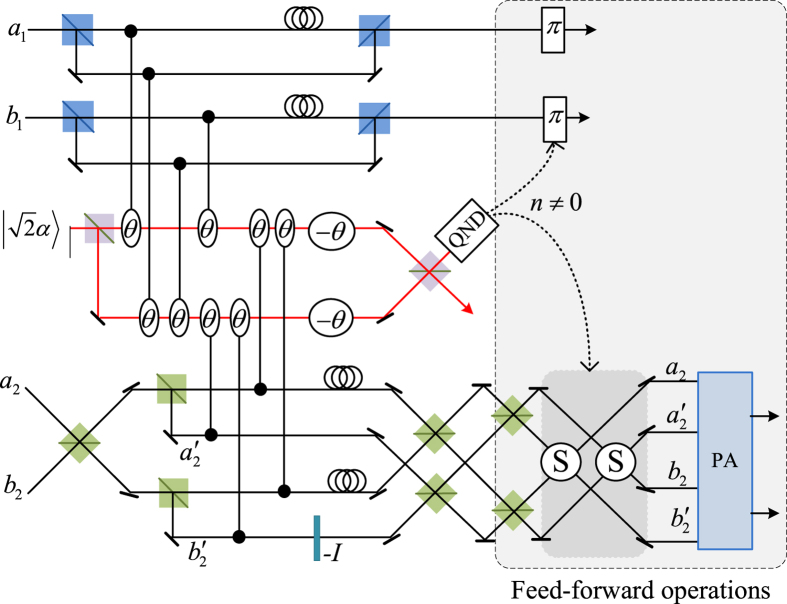
Schematic CNOT gate on a hybrid system consisting of the polarization DoF of one photon and the spatial DoF of the other photon. PS, BS, QND and PA are defined in the Fig. 1. −*I* and *S* are defined in the Fig. 2. An auxiliary probe beam is in the coherent state 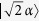.

Our consideration in this subsection is to realize a CNOT gate on the polarization DoF of one photon and the spatial DoF of the other. Schematic circuit is shown in Fig. 3. From the Figs 1 and 2, the photons *A*_1_ and *A*_2_ and the coherent photon will evolve as follows:


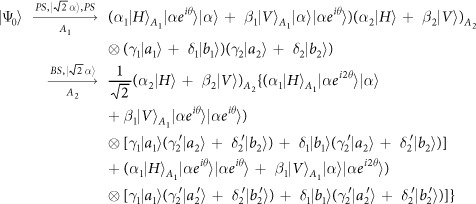



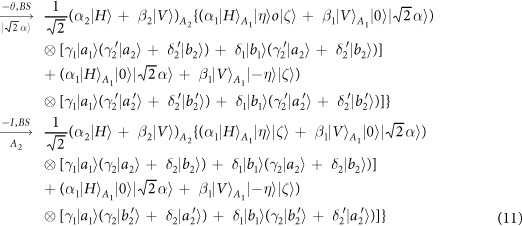


where 
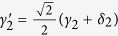
, 
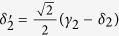
, |*η*〉 and |*ζ*〉 are defined in the Eq. (3).

Afterwards, the projection |*n*〉 〈*n*| is performed on the first qubus beam to get the proper output^49^,^51^. If the measurement outcome is *n* = 0, the photonic state in the Eq. (11) collapses into


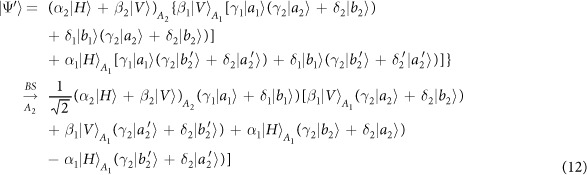


Now, from a PA for the photon *A*_2_, if *n* = 0 for the new Homodyne detection (the photon *A*_2_ passes through the modes *a*_2_ and *b*_2_), the state in the Eq. (12) will be


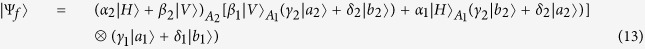


by switching the modes *a*_2_ and *b*_2_. Otherwise, *n* ≠ 0 for the new Homodyne detection (the photon *A*_2_ passes through the modes 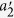 and 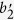), and the state in the Eq. (12) will be |Ψ_*f*_〉 in the Eq. (13) after performing a Pauli phase flip *Z* on the photon *A*_1_ and switching the modes 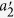 and 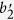.

If the measurement outcome satisfies *n* ≠ 0, the photonic state in the Eq. (11) collapses into


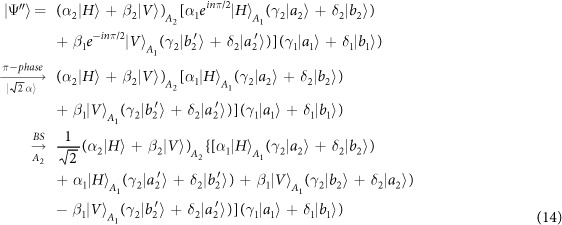


Now, from a PA for the photon *A*_2_, if *n* = 0 for the new Homodyne detection (the photon *A*_2_ passes through the modes *a*_2_ and *b*_2_), the state in the Eq. (14) will be |Ψ_*f*_〉 in the Eq. (13). If *n* ≠ 0 for the new Homodyne detection (the photon *A*_2_ passes through the modes 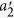 and 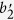), the state in the Eq. (14) will be |Ψ_*f*_〉 in the Eq. (13) after performing a Pauli phase flip *Z* on the photon *A*_1_. Thus a CNOT gate is nearly deterministically implemented on the polarization DoF of one photon and the spatial DoF of the other photon.

### CNOT gate on the hybrid spatial-polarization DoF of a two-photon system

**Figure 4:**
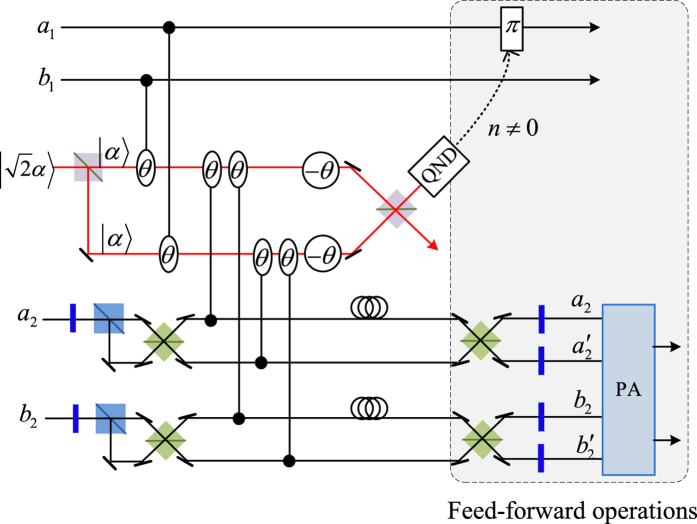
Schematic CNOT gate on the hybrid system consisting of the spatial DoF of one photon and the polarization DoF of the other photon. PS, BS, H, QND and PA are defined in the Fig. 1. An auxiliary probe beam is in the coherent state 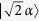.

Our consideration in this subsection is to realize a CNOT gate on the spatial DoF of one photon and the polarization DoF of the other. Schematic circuit is shown in Fig. 4. From the Figs 1 and 2, two photons *A*_1_ and *A*_2_ and the coherent pulse will evolve as follows:


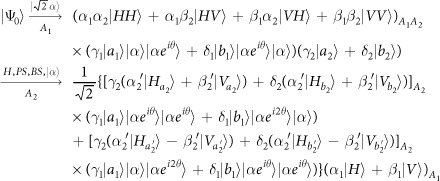



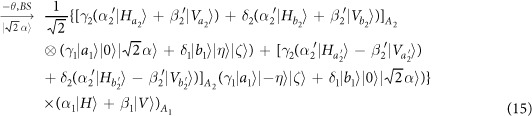


where 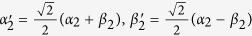, |*η*〉 and |*ζ*〉 are defined in the Eq. (3).

Afterwards, the projection |*n*〉 〈*n*| is performed on the first qubus beam to get the proper output^53^. If the measurement outcome is *n* = 0, the photonic state in the Eq. (15) collapses into


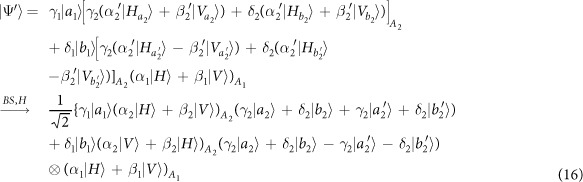


Now, from a PA for the photon *A*_2_, if *n* = 0 for the new Homodyne detection (the photon *A*_2_ passes through the modes *a*_2_ and *b*_2_), the state in the Eq. (16) will be


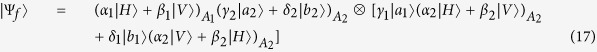


Otherwise, *n* ≠ 0 for the new Homodyne detection (the photon *A*_2_ passes through the modes *a*′_2_ and 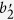), and the state in the Eq. (16) will be |Ψ_*f*_〉 in the Eq. (17) after −*I* being performed on the photon *A*_1_ from the mode *b*_1_.

If the measurement outcome satisfies *n* ≠ 0, the photonic state in the Eq. (15) collapses into


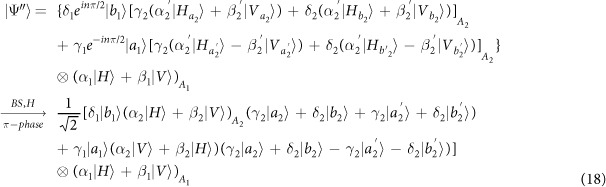


Now, from a PA for the photon *A*_2_, if *n* = 0 for the new Homodyne detection (the photon *A*_2_ passes through the modes *a*_2_ and *b*_2_), the state in the Eq. (18) will be |Ψ_*f*_〉 in the Eq. (17). Otherwise, *n* ≠ 0 for the new Homodyne detection (the photon *A*_2_ passes through the modes 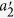 and 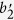), and the state in the Eq. (18) will be |Ψ_*f*_〉 in the Eq. (17) after −*I* being performed on the photon *A*_1_ from the mode *a*_1_. Thus a CNOT gate is nearly deterministically implemented on the hybrid system consisted of the spatial DoF of one photon and the polarization DoF of the other photon.

### CNOT gate on one photon with two DoFs

**Figure 5:**
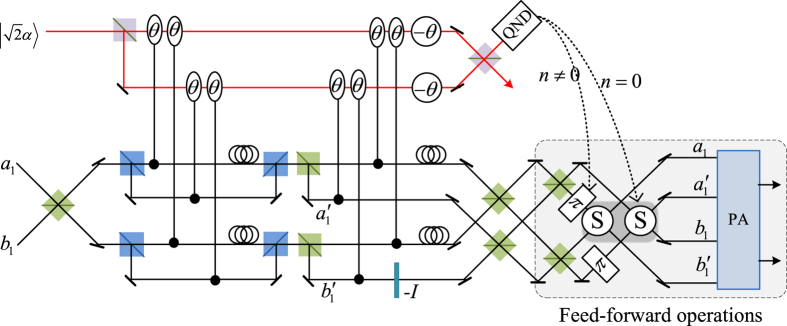
Schematic CNOT gate on one photon with two DoFs. PS, BS, QND and PA are defined in the Fig. 1. −*I* and S are defined in the Fig. 2. An auxiliary probe beam is in the coherent state 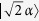.

Our considerations in this subsection is to realize a CNOT gate on one photon *A*_1_ with two DoFs. It is trivial to realize a CNOT gate when the spatial DoF of one photon is the controlling qubit. For the polarization DoF as the controlling qubit, its schematic circuit is shown in Fig. 5. In detail, the photon *A*_1_ and the coherent photon will evolve as follows:


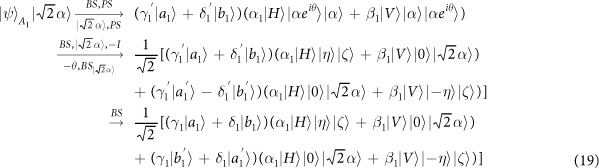


where 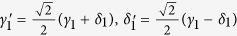, |*ζ*〉 are defined in the Eq. (3), and 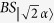 denotes the BS for coherent photons defined in the Fig. 1.

Afterwards, the projection |*n*〉 〈*n*| is performed on the first qubus beam to get the proper output^53^. If the measurement outcome is *n* = 0, the photonic state in the Eq. (19) collapses into


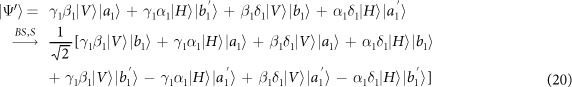


After a PA for the photon *A*_1_, if *n* = 0 for the new Homodyne detection (the photon *A*_1_ passes through the modes *a*_1_ and *b*_1_), the state in the Eq. (20) will be


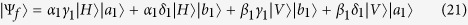


Otherwise, *n* ≠ 0 for the new Homodyne detection (the photon *A*_1_ passes through the modes 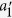 and 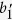), and the state *A*_1_ in the Eq. (20) will be |Ψ_*f*_〉 in the Eq. (21) after *Z* being performed on the photon *A*_1_.

If the measurement outcome satisfies *n* ≠ 0, the photonic state in the Eq. (19) collapses into


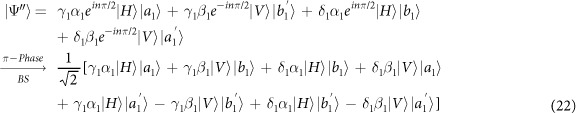


After a PA for the photon *A*_1_, the state in the Eq. (22) will be |Ψ_*f*_〉 in the Eq. (21) if *n* = 0 for the new Homodyne detection (the photon *A*_1_ passes through the modes *a*_1_ and *b*_1_). If *n* ≠ 0 for the new Homodyne detection (the photon *A*_1_ passes through the modes 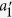 and 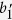), the state in the Eq. (22) will be |Ψ_*f*_〉 in the Eq. (21) after *Z* being performed on the photon *A*_1_.

### Quantum teleportation assisted by the weak cross-Kerr nonlinearity

**Figure 6:**
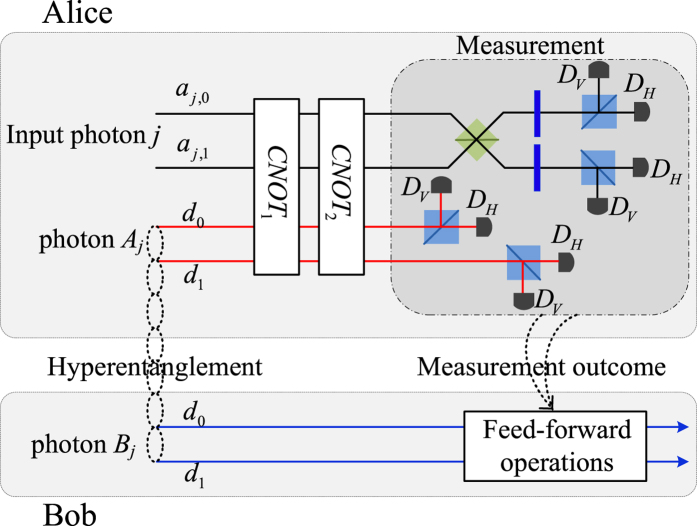
Schematic teleportation of arbitrary *n*-photon system with two DoFs. The quantum channel is constructed by hyperentanglements 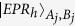, *j* = 1, ..., *n. X*^*p*^ = |0〉 〈1| + |1〉 〈0| and *X*^*s*^ = |*r*_1_〉 〈*r*_2_| + |*r*_2_〉 〈*r*_1_| denote Pauli flips on the polarization DoF and spatial DoF {*r*_1_, *r*_2_} of one photon, respectively. *CNOT*_1_ = |0〉 〈0| ⊗ *I*_2_ + |1〉 〈1| ⊗ *X*^*p*^ and *CNOT*_2_ = |*d*_0_〉 〈*d*_0_| ⊗ *I*_2_ + |*d*_1_〉 〈*d*_1_| ⊗ *X*^*s*^ are preformed on the photon *A*_*j*_ and the input photon *j*. PS, BS and H-plate are defined in the Fig. 1. *D*_*H*_ and *D*_*V*_ are single photon detectors. The feed-forward operations of Bob are only single photon operations.

Suppose that Alice wants to teleport an arbitrary *n*-photon system in the state


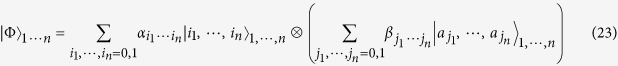


to Bob, where 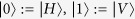, *a*_*k*,0_ and *a*_*k*,1_ denote the spatial modes of the input photon *k*. The quantum channel is constructed by hyperentanglements^32^


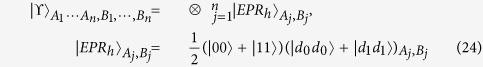


where the photons *A*_1_, ..., *A*_*n*_ belong to Alice while the photons *B*_1_, ..., *B*_*n*_ own to Bob. For special case of *n* = 1, Wang *et al*.^32^ have experimentally teleported a photon with the spin angular momentum and orbital angular momentum DoFs while Graham *et al*.^33^ teleported a specific photon of two DoFs with only phase information. Sheng *et al*.^44^ have proposed a theoretical teleportation using the Bell analysis assisted by the cross-Ker nonlinearity. Luo *et al*.^64^ have proposed a general teleportation of hybrid two-qubit systems assisted by the QED-cavity nonlinearity. In this subsection, by using present CNOT gates, we can complete the teleportation task with arbitrary *n* ≥ 1. Schematic circuit is shown in Fig. 6. These photons evolve as follows


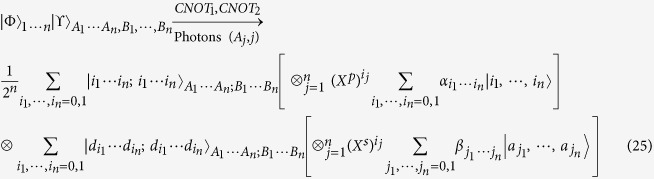


where *CNOT*_1_ = |0〉 〈0| ⊗ *I*_2_ + |1〉 〈1| ⊗ *X*^*p*^ denotes a CNOT gate on the polarization DoFs of two photons and *CNOT*_2_ = |*d*_0_〉 〈*d*_0_| ⊗ *I*_2_ + |*d*_1_〉 〈*d*_1_| ⊗ *X*^*s*^ denotes a CNOT gate on the spatial DoFs of two photons. Now, by measuring each photon *j* = 1, ..., *n* under the basis {|*Ha*_*j*,0_〉, |*Ha*_*j*,1_〉, |*Va*_*j*,0_〉, |*Va*_*j*,1_〉} (using two PSs and four single photon detectors), and measuring each photon *A*_*j*_,*j* = 1, ..., *n* under the basis 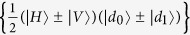 (using two *H*s, a *BS*, two *PS*s and four single photon detectors), each collapsed state of the photons *B*_1_, ..., *B*_*n*_ may be faithfully transferred into 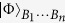 in the Eq. (23) by using local quantum single operations of Bob. Taking *n* = 2 as an example, all the collapsed states are shown in Table 1 of the Supplementary information with corresponding recovery operations.

### Quantum superdense coding assisted by the weak cross-Kerr nonlinearity

**Figure 7:**
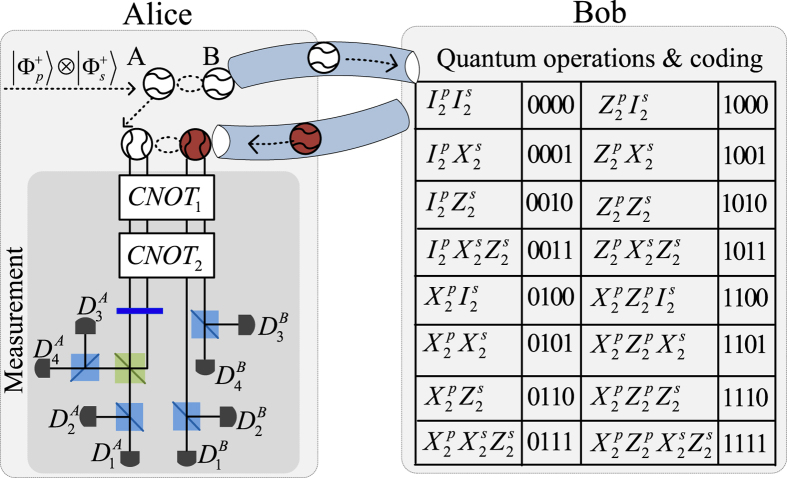
Schematic quantum superdense coding using the hyperentanglement in the Eq. (24). PS, BS and H are defined in the Fig. 1. *X*^*p*^, *X*^*s*^, *CNOT*_1_ and *CNOT*_2_ are defined in the Fig. 6. *Z*^*p*^ = |*H*〉 〈*H*| − |*V*〉 〈*V*| and *Z*^*s*^ = |*r*_1_〉 〈*r*_1_| − |*r*_2_〉 〈*r*_2_| denote the Pauli flip and Pauli phase flip on the spatial DoF {*r*_1_, *r*_2_} of one photon. 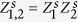, 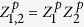, 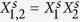, and 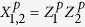. 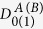 are single photon detectors. The feed-forward operations of Bob are only single photon operations.

Similarly, with the hyperentanglement, Alice and Bob may complete a general quantum superdense coding^65^,^66^, as shown in Fig. 7. Here, two photons *A* and *B* are prepared in 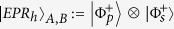 in the Eq. (24) by Alice. One photon *B* will be sent to Bob along Alice’s quantum channel. Now, Bob will perform a single photon operation on the received photon *B* according his coding of four bits *i*_1_*i*_2_*i*_3_*i*_4_ in the Fig. 7 and send back to Alice. The corresponding quantum measurements of Alice are shown in the Fig. 7. In detail, Alice performs two CNOT gates *CNOT*_1_ and *CNOT*_2_ on the photons *A* and *B*, let the output pulse of the photon *B* pass a H and a BS, and the photons *A* and *B* from each mode pass through a PS and be finally detected by single photon detectors. The resulting quantum hyperentanglements of Alice are shown in Table 2 of the Supplementary information. Different from previous quantum superdense coding which has realized two bits per photon transmission^60^,^61^, four bits can be communicated by sending a single photon.

### Quantum computation assisted by the weak cross-Kerr nonlinearity

Previous schemes have shown that the controlled logic gates may be performed on the polarization state using the spatial DoF as auxiliary quantum resources^12^,^13^,^14^,^22^,^23^. Although it is easy to switch different DoFs of one photon if only one DoF is used to encode information in quantum application, their conversions may cause confusions when two DoFs or more DoFs are independently used for encoding different information in one quantum task. With the present CNOT gates assisted by the weak cross-Kerr nonlinearity, the polarization and spatial DoFs of photonic states can be used as independent qubits without auxiliary DoFs. It means that two DoFs of each photon may be used as encoding qubits or register qubits simultaneously. In this case, the simulation resources may be saved one half. This may be very important for large-scale simulations such as the Shor algorithm. To show the implementation complexity of our CNOT gates, the comparisons of these CNOT gates with previous photonic implementations are shown in Table 1. All the linear optical elements of wave plates [H, Z, -I] and beam splitters [BS and PS] may be ignored because of their simplicities. It means that the complexity mainly depends of the cross-phase modulation, the interferences, and ancillary photons. From this table, the most of photonic CNOT gates with two DoFs [except *CNOT*_*s*,*p*,1_ using one wave plate] should involve more interactions with the weak-Ker nonlinearity than other schemes with single DoF^22^,^23^,^51^,^52^,^53^. The main difference is derived from an additional DoF in comparison with previous single DoF. In experiment, the added complexity may be reasonable because the perfect single photon is difficult and expensive with the modern physic technique. Using the photon number non-resolving detector for PND, ancillary single photons are avoided for the QND^53^. If this efficient way^53^ is used for our QNDs ancillary photons are not required in our CNOT gates, which are different from the qubus mediated CNOT gate in ref. 22. Moreover, the DXPM method^50^,^51^,^52^,^53^ are explored in our schemes to avoid the impractical XPM with a shift −*θ*^21^,^22^,^23^. Compared with the scheme in ref. 21, our schemes donot require displacement operations on the qubus beams, which may be hard to implement for large displacement amplitudes. Besides, coherent resources are necessary in all schemes and may be recycled. The complexity of the circuit in ref. 52 is same as these for general two-qubit gates. Generally, one may trade off the implementation complexity and simulation resources by choosing proper photon systems with one DoF and two DoFs.

## Discussions and Conclusions

The present CNOT gates on photons with two DoFs may be nearly deterministically performed. These CNOT gates are different from CNOT gates on photonic systems with only one DoF^11^,^12^,^13^,^14^,^15^,^22^,^23^,^24^,^25^,^26^,^27^,^28^,^29^, where the latter is always applied in quantum applications using the polarization DoF while other DoFs such as the momentum and time-bin are not considered or only considered as auxiliary systems^11^,^15^,^22^,^23^. Our CNOT gates show that quantum tasks may be simulated using photonic systems with two DoFs assisted by the weak cross-Kerr nonlinearity. During simulations, each DoF of one photon can be encoded as an independent qubit for storing or transferring quantum information. The key elements are the present CNOT gates which provide us useful primitives to manipulate photons with two DoFs.

Up to now, a well cross-Kerr nonlinearity in the optical single-photon regime is still difficult with current technology even lots of related results have been obtained^67^. In fact, Kok *et al*.^68^ showed that the Kerr phase shift is only *τ* ≈ 10^−18^ to operate in the optical single-photon regime. It may be improved to *τ* ≈ 10^−5^ using electromagnetically induced transparent materials. Recently, Gea-Banacloche^69^ shows that it is impossible to obtain large phase shifts via the giant Kerr effect with single-photon wave packets, as pointed out in refs 70,71. Note that −*θ* is indeed a large phase shift *π*/2 − *θ*. The weak cross-Kerr nonlinearity will make the phase shift ±*θ* of the coherent state become extremely small^72^. To address this problem, we take use of the double cross-phase modulation method^49^,^51^,^52^,^53^ to avoid the impractical −*θ*. Combining with a photon-number-resolving (PNR) detector, a homodyne detector may be used to discriminate two coherent states^53^,^73^. The post-selection strategy is useful in order to lower the error probability. PNR has been realized at infrared wavelengths, operating at room temperature and with a large dynamic range^74^, or at an operating wavelength of about 850 nm^75^. New measurement scheme has been realized based on a displacement operation followed by a PNR^76^. PNR has also been discussed with integrated optical circuit in the telecom band at 1550 nm based on UV-written silica-on-silicon waveguides and modified transition-edge sensors^77^. Of course, the PNR capability may be also shown from InGaAs single photon avalanche detectors, arrays of silicon photomultipliers, transition edge sensors and InGaAs with self-differencing circuits. Recently, superconducting nanowire as another candidate may provide free-running single-photon sensitivity from visible to mid-infrared frequencies, low dark counts, excellent timing resolution and short dead time, at an easily accessible temperature. Myoren *et al*. demonstrate the superconducting nanowire single-photon detectors with series-parallel meander-type configurations to have photon-number-resolving capabilities^78^. Some methods and device configurations are also proposed to obtain PNR capability using superconducting nanowire detectors^79^. By exploiting a superconducting qubit Lecocq *et al*. measure the photon/phonon-number distributions during these optomechanical interactions which may provide an essential non-linear resource^80^. Moreover, Weng *et al*. take use of quantum dot coupled resonant tunneling diodes to demonstrate a PNR^81^. Proposed electron-injecting operation may turn photon-switches to OFF state and make the detector ready for multiple-photons detection. Their results showed that the new PNR is better than a homodyne receiver. Hence, the present CNOT gates may be feasible if we choose a suitable Kerr nonlinear media and some good quantum measurement strategies on coherent beams.

In conclusion, we have proposed the parallel quantum computation based on two DoFs of photon systems, without auxiliary spatial or polarization DoFs. We have constructed five nearly deterministic CNOT gates (except one trivial CNOT gate) operating on the spatial and polarization DoFs of the two-photon system or one-photon system. With these CNOT gates, two DoFs of each photon may be independently encoded as different qubits in each task. We also discussed their applications of the quantum teleportation, quantum supertense coding and quantum computation. We concluded that one can teleport arbitrary *n*-photon in two DoFs when the hyperentanglement channels are set up and present CNOT gates are permitted perfectly. Moreover, we have obtained new quantum supertense coding in which a hyperentanglement is used to transfer four bits per photon transmission. For different quantum computation tasks, one may perform their simulations using photonic systems with two DoFs. In this case, quantum simulation resources are reduced to one half. All these results may be useful in various quantum applications.

## Methods

### The weak cross-Kerr nonlinearity

The cross-Kerr nonlinearity^21^,^22^,^23^ has a Hamiltonian in the form 
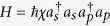
. Here, 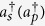 and *a*_*s*_(*a*_*p*_) represent the creation and annihilation operations, respectively, and the subscript *s*(*p*) denotes the signal (probe) mode. *χ* is the coupling strength of the nonlinearity decided by the cross-Kerr medium. Given a signal field |*n*_*a*_〉 and a probe beam |*α*〉, after photons passing through the cross-Kerr medium, the joint state of the combined system will be


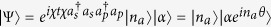


where *θ* = *χt* and *t* is the interaction time. Thus, by measuring the phase of the probe beam, the photon numbers may be distinguished in the signal mode, that is, the state |Ψ〉 will project into a number state.

### The parity gate

To distinguish different outputs of one photon with four modes, a parity gate (PA) is used using an ancillary coherent state 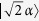, see the Fig. 1. The detailed evolution is defined as follows for the any initial system


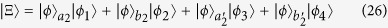


where 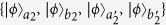 are different states of the photon *A*_2_ with four spatial modes *a*_2_, *b*_2_, 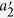 and 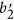, while {|*ϕ*_1_〉, |*ϕ*_2_〉, |*ϕ*_3_〉, |*ϕ*_4_〉} are corresponding states of the other system except the photon *A*_2_. In detail, the photon *A*_2_ from the modes 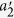 and 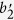 is firstly interacted with the coherent system in order. One can get a joint system


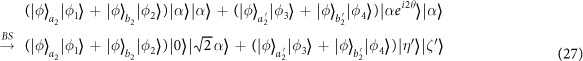


where 
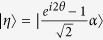
 and 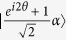. Afterwards, the projection |*n*〉 〈*n*| is performed on the first qubus beam to get the proper output^49^,^51^. If the measurement outcome is *n* = 0, the photonic state in the Eq. (27) collapses into


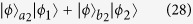


If the measurement outcome satisfies *n* ≠ 0, the photonic state in the Eq. (27) collapses into


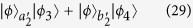


## Additional Information

**How to cite this article**: Luo, M.-X. *et al*. Quantum computation based on photonic systems with two degrees of freedom assisted by the weak cross-Kerr nonlinearity. *Sci. Rep.* **6**, 29939; doi: 10.1038/srep29939 (2016).

## Supplementary Material

Supplementary Information

## Figures and Tables

**Table 1:** The comparisons of our CNOT gates with previous photonic implementations.

Type	*n*_*i*_	*n*_*p*_	*n*_*QND*_	|*ϕ*_*a*_〉	*L*	*Ps*
*CNOT*_*p*,*p*_ in ref. 22	4	2	2	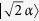	XPM	≈1
*CNOT*_*p*,*p*_ in ref. 23	2	0	1	|*α*〉	XPM	1/2
*CNOT*_*p*,*s*_ in ref. 51	4	0	1	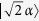	DXPM	≈1
*CNOT*_*p*,*p*_ in ref. 52	12	1	9	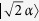	DXPM	≈1
*CNOT*_*p*,*p*_ in ref. 53	5	0	2	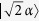	DXPM	≈1
*CNOT*_*p*,*p*_ in Fig. 1	8	0	2	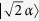	DXPM	≈1
*CNOT*_*s*,*s*_ in Fig. 2	8	0	2	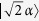	DXPM	≈1
*CNOT*_*s*,*p*_ in Fig. 3	10	0	2	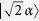	DXPM	≈1
*CNOT*_*p*,*s*_ in Fig. 4	8	0	2	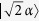	DXPM	≈1
*CNOT*_*p*,*s*,1_ in Fig. 5	10	0	2	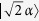	DXPM	≈1

*P*_*s*_ denotes the success probability. *CNOT*_*p*,*p*_ denotes the CNOT gate on the polarization DoF of two photons. *CNOT*_*p*,*s*,1_ denotes the polarization DoF and spatial mode of one photon. *CNOT*_*p*,*s*_ denotes the polarization DoF of one photon and the spatial mode of the other photon. *CNOT*_*s*,*p*_ denotes the spatial DoF of one photon and the polarization DoF of the other photon. *CNOT*_*s*,*s*_ denotes the spatial DoFs of two photons. *n*_*i*_ denotes the number of the interaction between the input photon and a coherent state with the cross-Kerr nonlinearities. *n*_*p*_ denotes the number of ancillary photons. *n*_*QND*_ denotes the number of the QND. |*ϕ*_*a*_〉 denotes the auxiliary coherent state. *L* ∈ {*DXPM, XPM*} denotes the double cross-phase modulation technique or cross-phase modulation technique. *CNOT*_*p*,*p*_ is easily followed by combining with the C-path gate and Merging gate^53^.
