# Supplementary material for: Quantum computation based on photonic systems with two degrees of freedom assisted by the weak cross-Kerr nonlinearity
Source: Sci Rep. 2016 Jul 18;6:29939. doi: 10.1038/srep29939 (PMC4947932; doi:10.1038/srep29939)
Supplement: Supplementary Information [file srep29939-s1.pdf]

Quantum computation based on photonic systems with two degrees of freedom assisted by the weak cross-Kerr nonlinearity

Ming-Xing Luo[1,2]*, Hui-Ran Li[1], Hong Lai[3]

[1] Information Security and National Computing Grid Laboratory,

Southwest Jiaotong University, Chengdu 610031, China,

[2] Department of Physics, University of Michigan, Ann Arbor, MI 48109, USA,

[2] School of Computer and Information Science, Southwest University, Chongqing 400715, China

June 12, 2016

*Corresponding author: mxluo@home.swjtu.edu.cn

Table 1: The relations between the detecting results of photon $1, 2, A_1, A_2$ and the feed-forward operations on two photons B_1 and B_2 for recovering two photons with two DoFs. $X^p = |0\rangle\langle 1| + |1\rangle\langle 0|$ and $Z^p = |0\rangle\langle 0| - |1\rangle\langle 1|$ denote Pauli flip and Pauli phase flip on polarization DoF of one photon, respectively, while $X^s = |r_1\rangle\langle r_2| + |r_2\rangle\langle r_1|$ and $Z^s = |r_1\rangle\langle r_1| - |r_2\rangle\langle r_2|$ denote Pauli flip and Pauli phase flip on spatial DoF $\{r_1, r_2\}$ of one photon, respectively.

	Photons A_1, A_2			
Photons $1, 2$	$\vert HHd_0d_0\rangle$	$\vert HHd_0d_1\rangle$	$\vert HHd_1d_0\rangle$	$\vert HHd_1d_1\rangle$
$\vert HHa_0a_0\rangle$	I	Z_2^s	Z_1^s	$Z_{1,2}^s$
$\vert HHa_0a_1\rangle$	X_2^s	$Z_2^sX_2^s$	$Z_1^sX_2^s$	$Z_{1,2}^sX_2^s$
$\vert HHa_1a_0\rangle$	X_1^s	$Z_2^sX_1^s$	$Z_1^sX_1^s$	$Z_{1,2}^sX_1^s$
$\vert HHa_1a_1\rangle$	$X_{1,2}^s$	$Z_2^sX_{1,2}^s$	$Z_1^sX_{1,2}^s$	$Z_{1,2}^sX_{1,2}^s$
$\vert HVa_0a_0\rangle$	X_2^p	$X_2^pZ_2^s$	$X_2^pZ_1^s$	$X_2^pZ_{1,2}^s$
$\vert HVa_0a_1\rangle$	$X_2^pX_2^s$	$X_2^pZ_2^sX_2^s$	$X_2^pZ_1^sX_2^s$	$X_2^pZ_{1,2}^sX_2^s$
$\vert HVa_1a_0\rangle$	$X_2^pX_1^s$	$X_2^pZ_2^sX_1^s$	$X_2^pZ_1^sX_1^s$	$X_2^pZ_{1,2}^sX_1^s$
$\vert HVa_1a_1\rangle$	$X_2^pX_{1,2}^s$	$X_2^pZ_2^sX_{1,2}^s$	$X_2^pZ_1^sX_{1,2}^s$	$X_2^pZ_{1,2}^sX_{1,2}^s$
$\vert VHa_0a_0\rangle$	X_1^p	$X_1^pZ_2^s$	$X_1^pZ_1^s$	$X_1^pZ_{1,2}^s$
$\vert VHa_0a_1\rangle$	$X_1^pX_2^s$	$X_1^pZ_2^sX_2^s$	$X_1^pZ_1^sX_2^s$	$X_1^pZ_{1,2}^sX_2^s$
$\vert VHa_1a_0\rangle$	$X_1^pX_1^s$	$X_1^pZ_2^sX_1^s$	$X_1^pZ_1^sX_1^s$	$X_1^pZ_{1,2}^sX_1^s$
$\vert VHa_1a_1\rangle$	$X_1^sX_1^sX_{1,2}^p$	$Z_2^sX_{1,2}^sX_{1,2}^p$	$Z_1^sX_{1,2}^sX_{1,2}^p$	$Z_{1,2}^sX_{1,2}^sX_{1,2}^p$
$\vert VVa_0a_0\rangle$	$X_{1,2}^p$	$Z_2^sX_{1,2}^p$	$Z_1^sX_{1,2}^p$	$Z_{1,2}^sX_{1,2}^p$
$\vert VVa_0a_1\rangle$	$X_2^sX_{1,2}^p$	$Z_2^sX_2^sX_{1,2}^p$	$Z_1^sX_2^sX_{1,2}^p$	$Z_{1,2}^sX_2^sX_{1,2}^p$
$\vert VVa_1a_0\rangle$	$X_1^sX_{1,2}^p$	$Z_2^sX_1^sX_{1,2}^p$	$Z_1^sX_1^sX_{1,2}^p$	$Z_{1,2}^sX_1^sX_{1,2}^p$
$\vert VVa_1a_1\rangle$	$X_{1,2}^sX_{1,2}^p$	$Z_2^sX_{1,2}^sX_{1,2}^p$	$Z_1^sX_{1,2}^sX_{1,2}^p$	$Z_{1,2}^sX_{1,2}^sX_{1,2}^p$
	$\vert HVd_0d_0\rangle$	$\vert HVd_0d_1\rangle$	$\vert HVd_1d_0\rangle$	$\vert HVd_1d_1\rangle$
$\vert HHa_0a_0\rangle$	Z_2^p	$Z_2^sZ_2^p$	$Z_1^sZ_2^p$	$Z_{1,2}^sZ_2^p$
$\vert HHa_0a_1\rangle$	$Z_2^pX_2^s$	$Z_2^sZ_2^pX_2^s$	$Z_1^sZ_2^pX_2^s$	$Z_{1,2}^sZ_2^pX_2^s$
$\vert HHa_1a_0\rangle$	$Z_2^pX_1^s$	$Z_2^sZ_2^pX_1^s$	$Z_1^sZ_1^pX_1^s$	$Z_{1,2}^sZ_1^pX_1^s$
$\vert HHa_1a_1\rangle$	$Z_2^pX_{1,2}^s$	$Z_2^sZ_2^pX_{1,2}^s$	$Z_1^sZ_1^pX_{1,2}^s$	$Z_{1,2}^sZ_1^pX_{1,2}^s$
$\vert HVa_0a_0\rangle$	$Z_2^pX_2^p$	$Z_2^sZ_2^pX_2^p$	$Z_1^sZ_2^pX_2^p$	$Z_{1,2}^sZ_2^pX_2^p$
$\vert HVa_0a_1\rangle$	$Z_2^pX_2^sX_2^p$	$Z_2^sZ_2^pX_2^sX_2^p$	$Z_1^sZ_2^pX_2^sX_2^p$	$Z_{1,2}^sZ_2^pX_2^sX_2^p$
$\vert HVa_1a_0\rangle$	$Z_2^pX_1^sX_2^p$	$Z_2^sZ_2^pX_1^sX_2^p$	$Z_1^sZ_1^pX_1^sX_2^p$	$Z_{1,2}^sZ_1^pX_1^sX_2^p$
$\vert HVa_1a_1\rangle$	$Z_2^pX_{1,2}^sX_2^p$	$Z_2^sZ_2^pX_{1,2}^sX_2^p$	$Z_1^sZ_1^pX_{1,2}^sX_2^p$	$Z_{1,2}^sZ_1^pX_{1,2}^sX_2^p$
$\vert VHa_0a_0\rangle$	$Z_2^pX_1^p$	$Z_2^sZ_2^pX_1^p$	$Z_1^sZ_2^pX_1^p$	$Z_{1,2}^sZ_2^pX_1^p$
$\vert VHa_0a_1\rangle$	$Z_2^pX_2^sX_1^p$	$Z_2^sZ_2^pX_2^sX_1^p$	$Z_1^sZ_2^pX_2^sX_1^p$	$Z_{1,2}^sZ_2^pX_2^sX_1^p$
$\vert VHa_1a_0\rangle$	$Z_2^pX_1^sX_1^p$	$Z_2^sZ_2^pX_1^sX_1^p$	$Z_1^sZ_1^pX_1^sX_1^p$	$Z_{1,2}^sZ_1^pX_1^sX_1^p$
$\vert VHa_1a_1\rangle$	$Z_2^pX_{1,2}^sX_1^p$	$Z_2^sZ_2^pX_{1,2}^sX_1^p$	$Z_1^sZ_1^pX_{1,2}^sX_1^p$	$Z_{1,2}^sZ_1^pX_{1,2}^sX_1^p$
$\vert VVa_0a_0\rangle$	$Z_2^pX_{1,2}^p$	$Z_2^sZ_2^pX_{1,2}^p$	$Z_1^sZ_2^pX_{1,2}^p$	$Z_{1,2}^sZ_2^pX_{1,2}^p$
$\vert VVa_0a_1\rangle$	$Z_2^pX_2^sX_{1,2}^p$	$Z_2^sZ_2^pX_2^sX_{1,2}^p$	$Z_1^sZ_2^pX_2^sX_{1,2}^p$	$Z_{1,2}^sZ_2^pX_2^sX_{1,2}^p$
$\vert VVa_1a_0\rangle$	$Z_2^pX_1^sX_{1,2}^p$	$Z_2^sZ_2^pX_1^sX_{1,2}^p$	$Z_1^sZ_1^pX_1^sX_{1,2}^p$	$Z_{1,2}^sZ_1^pX_1^sX_{1,2}^p$
$\vert VVa_1a_1\rangle$	$Z_2^pX_{1,2}^sX_{1,2}^p$	$Z_2^sZ_2^pX_{1,2}^sX_1^pX_1^p$	$Z_1^sZ_1^pX_{1,2}^sX_{1,2}^p$	$Z_{1,2}^sZ_1^pX_{1,2}^sX_{1,2}^p$

Continuing of Table I.

	Photons A_1, A_2			
Photons $1, 2$	$\vert VHd_0d_0\rangle$	$\vert VHd_0d_1\rangle$	$\vert VHd_1d_0\rangle$	$\vert VHd_1d_1\rangle$
$\vert HHa_0a_0\rangle$	Z_1^p	$Z_2^sZ_1^p$	$Z_1^sZ_1^p$	$Z_{1,2}^sZ_1^p$
$\vert HHa_0a_1\rangle$	$Z_1^pX_2^s$	$Z_2^sZ_1^pX_2^s$	$Z_1^sZ_1^pX_2^s$	$Z_{1,2}^sZ_1^pX_2^s$
$\vert HHa_1a_0\rangle$	$Z_1^pX_1^s$	$Z_2^sZ_1^pX_1^s$	$Z_1^sZ_1^pX_1^s$	$Z_{1,2}^sZ_1^pX_1^s$
$\vert HHa_1a_1\rangle$	$Z_1^pX_{1,2}^s$	$Z_2^sZ_1^pX_{1,2}^s$	$Z_1^sZ_1^pX_{1,2}^s$	$Z_{1,2}^sZ_1^pX_{1,2}^s$
$\vert HVa_0a_0\rangle$	$Z_1^pX_2^p$	$Z_2^sZ_1^pX_2^p$	$Z_1^sZ_1^pX_2^p$	$Z_{1,2}^sZ_1^pX_2^p$
$\vert HVa_0a_1\rangle$	$Z_1^pX_2^sX_2^p$	$Z_2^sZ_1^pX_2^sX_2^p$	$Z_1^sZ_1^pX_2^sX_2^p$	$Z_{1,2}^sZ_1^pX_2^sX_2^p$
$\vert HVa_1a_0\rangle$	$Z_1^pX_1^sX_2^p$	$Z_2^sZ_1^pX_1^sX_2^p$	$Z_1^sZ_1^pX_1^sX_2^p$	$Z_{1,2}^sZ_1^pX_1^sX_2^p$
$\vert HVa_1a_1\rangle$	$Z_1^pX_2^sX_1^sX_2^p$	$Z_2^sZ_1^pX_{1,2}^sX_2^p$	$Z_1^sZ_1^pX_{1,2}^sX_2^p$	$Z_{1,2}^sZ_1^pX_{1,2}^sX_2^p$
$\vert VHa_0a_0\rangle$	$Z_1^pX_1^p$	$Z_2^sZ_1^pX_1^p$	$Z_1^sZ_1^pX_1^p$	$Z_{1,2}^sZ_1^pX_1^p$
$\vert VHa_0a_1\rangle$	$Z_1^pX_2^sX_1^p$	$Z_2^sZ_1^pX_2^sX_1^p$	$Z_1^sZ_1^pX_2^sX_1^p$	$Z_{1,2}^sZ_1^pX_2^sX_1^p$
$\vert VHa_1a_0\rangle$	$Z_1^pX_1^sX_1^p$	$Z_2^sZ_1^pX_1^sX_1^p$	$Z_1^sZ_1^pX_1^sX_1^p$	$Z_{1,2}^sZ_1^pX_1^sX_1^p$
$\vert VHa_1a_1\rangle$	$Z_1^pX_{1,2}^sX_1^p$	$Z_2^sZ_1^pX_{1,2}^sX_1^p$	$Z_1^sZ_1^pX_{1,2}^sX_1^p$	$Z_{1,2}^sZ_1^pX_{1,2}^sX_1^p$
$\vert VVa_0a_0\rangle$	$Z_1^pX_{1,2}^p$	$Z_2^sZ_1^pX_{1,2}^p$	$Z_1^sZ_1^pX_{1,2}^p$	$Z_{1,2}^sZ_1^pX_{1,2}^p$
$\vert VVa_0a_1\rangle$	$Z_1^pX_2^sX_{1,2}^p$	$Z_2^sZ_1^pX_2^sX_{1,2}^p$	$Z_1^sZ_1^pX_2^sX_{1,2}^p$	$Z_{1,2}^sZ_1^pX_2^sX_{1,2}^p$
$\vert VVa_1a_0\rangle$	$Z_1^pX_1^sX_{1,2}^p$	$Z_2^sZ_1^pX_1^sX_{1,2}^p$	$Z_1^sZ_1^pX_1^sX_{1,2}^p$	$Z_{1,2}^sZ_1^pX_1^sX_{1,2}^p$
$\vert VVa_1a_1\rangle$	$Z_1^pX_{1,2}^sX_{1,2}^p$	$Z_2^sZ_1^pX_{1,2}^sX_{1,2}^p$	$Z_1^sZ_1^pX_{1,2}^sX_{1,2}^p$	$Z_{1,2}^sZ_1^pX_{1,2}^sX_{1,2}^p$
	$\vert VVd_0d_0\rangle$	$\vert VVd_0d_1\rangle$	$\vert VVd_1d_0\rangle$	$\vert VVd_1d_1\rangle$
$\vert HHa_0a_0\rangle$	$Z_{1,2}^p$	$Z_2^sZ_{1,2}^p$	$Z_1^sZ_{1,2}^p$	$Z_{1,2}^sZ_{1,2}^p$
$\vert HHa_0a_1\rangle$	$Z_{1,2}^pX_2^s$	$Z_2^sZ_{1,2}^pX_2^s$	$Z_1^sZ_{1,2}^pX_2^s$	$Z_{1,2}^sZ_{1,2}^pX_2^s$
$\vert HHa_1a_0\rangle$	$Z_{1,2}^pX_1^s$	$Z_2^sZ_{1,2}^pX_1^s$	$Z_1^sZ_{1,2}^pX_1^s$	$Z_{1,2}^sZ_{1,2}^pX_1^s$
$\vert HHa_1a_1\rangle$	$Z_{1,2}^pX_{1,2}^s$	$Z_2^sZ_{1,2}^pX_{1,2}^s$	$Z_1^sZ_{1,2}^pX_{1,2}^s$	$Z_{1,2}^sZ_{1,2}^pX_{1,2}^s$
$\vert HVa_0a_0\rangle$	$Z_{1,2}^pX_2^p$	$Z_2^sZ_{1,2}^pX_2^p$	$Z_1^sZ_{1,2}^pX_2^p$	$Z_{1,2}^sZ_{1,2}^pX_2^p$
$\vert HVa_0a_1\rangle$	$Z_{1,2}^pX_2^sX_2^p$	$Z_2^sZ_{1,2}^pX_2^sX_2^p$	$Z_1^sZ_{1,2}^pX_2^sX_2^p$	$Z_{1,2}^sZ_{1,2}^pX_2^sX_2^p$
$\vert HVa_1a_0\rangle$	$Z_{1,2}^pX_1^sX_2^p$	$Z_2^sZ_{1,2}^pX_1^sX_2^p$	$Z_1^sZ_{1,2}^pX_1^sX_2^p$	$Z_{1,2}^sZ_{1,2}^pX_1^sX_2^p$
$\vert HVa_1a_1\rangle$	$Z_{1,2}^pX_{1,2}^sX_2^p$	$Z_2^sZ_{1,2}^pX_{1,2}^sX_2^p$	$Z_1^sZ_{1,2}^pX_{1,2}^sX_2^p$	$Z_{1,2}^sZ_{1,2}^pX_{1,2}^sX_2^p$
$\vert VHa_0a_0\rangle$	$Z_{1,2}^pX_1^p$	$Z_2^sZ_{1,2}^pX_1^p$	$Z_1^sZ_{1,2}^pX_1^p$	$Z_{1,2}^sZ_{1,2}^pX_1^p$
$\vert VHa_0a_1\rangle$	$Z_{1,2}^pX_2^sX_1^p$	$Z_2^sZ_{1,2}^pX_2^sX_1^p$	$Z_1^sZ_{1,2}^pX_2^sX_1^p$	$Z_{1,2}^sZ_{1,2}^pX_2^sX_1^p$
$\vert VHa_1a_0\rangle$	$Z_{1,2}^pX_1^sX_1^p$	$Z_2^sZ_{1,2}^pX_1^sX_1^p$	$Z_1^sZ_{1,2}^pX_1^sX_1^p$	$Z_{1,2}^sZ_{1,2}^pX_1^sX_1^p$
$\vert VHa_1a_1\rangle$	$Z_{1,2}^pX_{1,2}^sX_1^p$	$Z_2^sZ_{1,2}^pX_{1,2}^sX_1^p$	$Z_1^sZ_{1,2}^pX_{1,2}^sX_1^p$	$Z_{1,2}^sZ_{1,2}^pX_{1,2}^sX_1^p$
$\vert VVa_0a_0\rangle$	$Z_{1,2}^pX_{1,2}^p$	$Z_2^sZ_{1,2}^pX_{1,2}^p$	$Z_1^sZ_{1,2}^pX_{1,2}^p$	$Z_{1,2}^sZ_{1,2}^pX_{1,2}^p$
$\vert VVa_0a_1\rangle$	$Z_{1,2}^pX_2^sX_{1,2}^p$	$Z_2^sZ_{1,2}^pX_2^sX_{1,2}^p$	$Z_1^sZ_{1,2}^pX_2^sX_{1,2}^p$	$Z_{1,2}^sZ_{1,2}^pX_2^sX_{1,2}^p$
$\vert VVa_1a_0\rangle$	$Z_{1,2}^pX_1^sX_{1,2}^p$	$Z_2^sZ_{1,2}^pX_1^sX_{1,2}^p$	$Z_1^sZ_{1,2}^pX_1^sX_{1,2}^p$	$Z_{1,2}^sZ_{1,2}^pX_1^sX_{1,2}^p$
$\vert VVa_1a_1\rangle$	$Z_{1,2}^pX_{1,2}^sX_{1,2}^p$	$Z_2^sZ_{1,2}^pX_{1,2}^sX_{1,2}^p$	$Z_1^sZ_{1,2}^pX_{1,2}^sX_{1,2}^p$	$Z_{1,2}^sZ_{1,2}^pX_{1,2}^sX_{1,2}^p$

Table 2: Quantum superdense coding with hyperentanglement.

Transferred bits	Bob's operation	Resulting two-photon state	Alice's measurement
0000	$I_2^p I_2^s$	$\frac{1}{2}(\|HH\rangle + \|VV\rangle)(\|a_1a_1\rangle + \|b_1b_1\rangle$	$D_1^A D_1^B := \|HHa_1a_1\rangle$
0001	$I_2^p X_2^s$	$\frac{1}{2}(\|HH\rangle + \|VV\rangle)(\|a_1b_1\rangle + \|b_1a_1\rangle)$	$D_1^A D_2^B := \|HHa_1b_1\rangle$
0010	$I_2^p Z_2^s$	$\frac{1}{2}(\|HH\rangle + \|VV\rangle)(\|a_1a_1\rangle - \|b_1b_1\rangle)$	$D_1^A D_3^B := \|HHb_1a_1\rangle$
0011	$I_2^p Z_2^s X_2^s$	$\frac{1}{2}(\|HH\rangle + \|VV\rangle)(\|a_1b_1\rangle - \|b_1a_1\rangle)$	$D_1^A D_4^B := \|HHb_1b_1\rangle$
0100	$X_2^p I_2^s$	$\frac{1}{2}(\|HV\rangle + \|VH\rangle)(\|a_1a_1\rangle + \|b_1b_1\rangle)$	$D_2^A D_1^B := \|HVa_1a_1\rangle$
0101	$X_2^p X_2^s$	$\frac{1}{2}(\|HV\rangle + \|VH\rangle)(\|a_1b_1\rangle + \|b_1a_1\rangle)$	$D_2^A D_2^B := \|HVa_1b_1\rangle$
0110	$X_2^p Z_2^s$	$\frac{1}{2}(\|HV\rangle + \|VH\rangle)(\|a_1a_1\rangle - \|b_1b_1\rangle)$	$D_2^A D_3^B := \|HVb_1a_1\rangle$
0111	$X_2^p Z_2^s X_2^s$	$\frac{1}{2}(\|HV\rangle + \|VH\rangle)(\|a_1b_1\rangle - \|b_1a_1\rangle)$	$D_2^A D_4^B := \|HVb_1b_1\rangle$
1000	$Z_2^p I_2^s$	$\frac{1}{2}(\|HH\rangle - \|VV\rangle)(\|a_1a_1\rangle + \|b_1b_1\rangle)$	$D_3^A D_1^B := \|VHa_1a_1\rangle$
1001	$Z_2^p X_2^s$	$\frac{1}{2}(\|HH\rangle - \|VV\rangle)(\|a_1b_1\rangle + \|b_1a_1\rangle)$	$D_3^A D_2^B := \|VHa_1b_1\rangle$
1010	$Z_2^p Z_2^s$	$\frac{1}{2}(\|HH\rangle - \|VV\rangle)(\|a_1a_1\rangle - \|b_1b_1\rangle)$	$D_3^A D_3^B := \|VHb_1a_1\rangle$
1011	$Z_2^p Z_2^s X_2^s$	$\frac{1}{2}(\|HH\rangle - \|VV\rangle)(\|a_1b_1\rangle - \|b_1a_1\rangle)$	$D_3^A D_4^B := \|VHb_1b_1\rangle$
1100	$Z_2^p X_2^p I_2^s$	$\frac{1}{2}(\|HV\rangle - \|VH\rangle)(\|a_1a_1\rangle + \|b_1b_1\rangle)$	$D_4^A D_1^B := \|VVa_1a_1\rangle$
1101	$Z_2^p X_2^p X_2^s$	$\frac{1}{2}(\|HV\rangle - \|VH\rangle)(\|a_1b_1\rangle + \|b_1a_1\rangle)$	$D_4^A D_2^B := \|VVa_1b_1\rangle$
1110	$Z_2^p X_2^p Z_2^s$	$\frac{1}{2}(\|HV\rangle - \|VH\rangle)(\|a_1a_1\rangle - \|b_1b_1\rangle)$	$D_4^A D_3^B := \|VVb_1a_1\rangle$
1111	$Z_2^p X_2^p Z_2^s X_2^s$	$\frac{1}{2}(\|HV\rangle - \|VH\rangle)(\|a_1b_1\rangle - \|b_1a_1\rangle)$	$D_4^A D_4^B := \|VVb_1b_1\rangle$
